# The Effect of Inulin on Lifespan, Related Gene Expression and Gut Microbiota in *InR*^p5545^/TM3 Mutant *Drosophila melanogaster*: A Preliminary Study

**DOI:** 10.3390/nu11030636

**Published:** 2019-03-15

**Authors:** Yuling Dong, Hao Sun, Weichao Yang, Shuang Ma, Beibei Du, Hui Xu

**Affiliations:** 1 Key Laboratory of Pollution Ecology and Environmental Engineering, Institute of Applied Ecology, Chinese Academy of Sciences, 72 Wenhua Road, Shenyang 110016, China; yuling-dong@163.com (Y.D.); haos@spaces.ac.cn (H.S.); yangweichao@iae.ac.cn (W.Y.); mas969@nenu.edu.cn (S.M.); dubeibeidb@163.com (B.D.); 2 University of Chinese Academy of Sciences, Beijing 100049, China

**Keywords:** inulin, *Drosophila melanogaster*, lifespan, insulin/insulin-like growth factor (IGF)-like signalling (IIS) pathway, gut microbiota

## Abstract

Inulin is considered an efficient prebiotic and is beneficial for metabolic diseases via promoting intestinal probiotic enrichment and the metabolites of short-chain fatty acids (SCFAs). However, the effect of inulin on patients with *InR* deficiencies has seldom been reported. In this study, the lifespan, related gene expression, and gut microbiota of *InR*^p5545^/TM3 (insulin receptor mutant) *Drosophila melanogaster* under inulin treatment were investigated. The results showed that the lifespan was extended in only males and not in females. Furthermore, distinctly different patterns of gene expression were found between males and females, especially in the insulin/insulin-like growth factor (IGF)-like signalling (IIS) and target of rapamycin (TOR) pathways. Additionally, as a link between inulin and lifespan responses, the gut microbiota was distinctly separated by gender in both the standard diet group and the inulin treatment group, and the relationship between lifespan and the gut microbiota community was stronger in male flies than in females. This study provides preliminary evidence for the gender-dependent lifespan responses to inulin in insulin signalling-deficient *Drosophila*. However, controls such as wild-type and TM3 flies, and more *InR* mutant strains with different genetic backgrounds need to be further investigated to elucidate the mechanisms underlying the phenomenon.

## 1. Introduction

Diet is considered an important factor associated with disease, especially certain nutritional metabolic disorders, such as obesity and diabetes [1,2]. The metabolites of food, especially those in high-fat, high-cholesterol and high-sugar diets, can directly or indirectly affect the physiological characteristics of the body and tend to lead to metabolic disorders [3,4,5]. Food metabolism in the body depends on not only the digestive organs but also on microorganisms in the digestive tract. Thus, in recent years, an increasing number of studies have focused on the role of digestive tract microorganisms and the relationship between gut microbiota and diseases [6,7,8].

Inulin, a well-known fermentable dietary fibre, relies heavily on the gut microbiota because it cannot be directly digested by the digestive tract [9,10]. Furthermore, inulin has been reported to have a beneficial effect on metabolic disease–type 2 diabetes via the regulation of circulating concentrations of free fatty acids (FFAs), blood glucose, and body mass [11]. The beneficial effects of inulin on the body manifest in mainly two ways: (1) inulin acts as an efficient prebiotic [12] by nourishing beneficial bacteria, such as *Bifidobacteria* [10] and *lactobacilli* [12], and enriching the short-chain fatty acids (SCFAs)-producing microbiota [13]; and (2) the SCFAs fermented by the gut microbiota play a key role in adipose, brain, and liver tissues as energy substrates, thereby reducing inflammation and tumourigenesis, improving insulin resistivity and AMPK activity, and reducing gluconeogenesis and lipid storage [14]. In addition, a previous study reported that inulin may regulate the production of peptides, including incretins, which are involved in the regulation of food intake and systemic effects [15].

*Drosophila* is a useful genetic model for research on metabolism and can be used to study the metabolic functions of the insulin/insulin-like growth factor (IGF)-like signalling (IIS) pathway because *Drosophila* shares the conserved IIS pathway with mammals [16]. As a link between insulin-like peptides (dIlps) and downstream effectors, the insulin receptor (*InR*) initiates a series of cascade events in IIS [16], and the *InR* mutant has been suggested to bear insulin signalling deficiency [17]. Inulin has also been shown to improve body iron status and alter the expression of genes involved in iron homeostasis and inflammation in young pigs [18]. However, the effect of inulin on lifespan has not been reported, and whether the related genes, especially IIS pathway genes, respond to inulin supplementation is still unknown. In this study, we investigated the effect of inulin on longevity, related gene expression, and gut bacterial community using *InR*^p5545^/TM3 mutant *Drosophila melanogaster* to reveal the interactions among inulin, probiotics, and related genes. The results showed a gender-dependent lifespan extension as well as distinct gene expression and intestinal bacterial community profiles in the two sex groups supplemented with inulin in *InR*^p5545^/TM3 mutant *Drosophila melanogaster*.

## 2. Materials and Methods

### 2.1. Fly Husbandry

This study used the *Drosophila melanogaster* stock *InR*^p5545^/TM3 (Bloomington Drosophila Stock Center #11661, Indiana University, Bloomington, IN, USA), generated by P-element insertion in exon 1, yielding nondwarf and viable flies [17]. The flies were raised on a standard yeast–sucrose–cornmeal diet containing 25 g yeast, 40 g sucrose, 42.4 g maltose, 66.825 g cornmeal, 9.18 g soybean meal, 6 g agar, 1 g sodium benzoate, 0.25 g nipagin, and 6.875 mL propionic acid per litre. The inulin treatment media comprised a standard diet supplemented with 5% inulin or 10% inulin (the inulin was kindly provided by Professor Yuetao Yi, Yantai Institute of Coastal Zone Research, Chinese Academy of Sciences). All flies were maintained under constant temperature (25 °C) and humidity (65%) with a 12 h light–dark cycle.

### 2.2. Survival Analysis

Flies incubated within 8 h were separated into single sex groups (males and females) under light CO_2_ anaesthesia. Ten vials containing 20 flies in each vial for both the male and female groups were used for survival analysis. Survival curves were determined by counting dead flies every 2–3 days, and the medium was replaced every 5 days.

For starvation assays, flies on the standard and inulin-supplemented diets were transferred to a vial containing phosphate buffered saline (PBS)/1% agar on day 20, and deaths were recorded at the intervals indicated in the graphs. Eight vials containing 20 flies each were counted for each condition. The body weights were measured using an ultrasensitive scale BSA223S (Sartorius, Beijing, China).

### 2.3. 16S Amplicon Analysis

On day 20, flies (from both the inulin and standard diet groups) were collected and sequentially rinsed in 50% (*v*/*v*) bleach and 70% ethanol, after which they were washed extensively with PBS before dissection. Guts from 15 flies in each sample were dissected in sterile PBS using sterile forceps, and the trachea, malpighian tubules, and crop were the carefully removed. Guts were collected in PBS on ice and then homogenized using a tissue grinder (Tiangen, OSE-Y20, Beijing, China) with a pestle (Tiangen, OSE-Y001, Beijing, China). Homogenized samples were stored at −80 °C, and frozen gut samples were thawed at 37 °C for 45 min in a 1.5 mL microcentrifuge tube and transferred to a 2 mL tube containing 600 μL LWA (BioBase, M2012-01, Chengdu, China). The supernatant was transferred to a 2 mL tube containing 30 μL BioBase Tissue beads (BioBase, M2012-01, Chengdu, China). After elution with WB and SPW buffers (BioBase, M2012-01, Chengdu, China), the beads were air-dried at room temperature. Finally, 200 μL of Buffer TL (BioBase, M2012-01, Chengdu, China) was added to each sample, followed by incubation at 56 °C for 15 min.

Genomic DNA was sent to GENEWIZ (Suzhou, China) for Illumina MiSeq sequencing. Sample-specific barcode sequences and sequencing adapters were added to the PCR amplicons for indexing. All indexed amplicons were pooled into one sequencing library; the integrity of the sequencing library was evaluated on a Bioanalyzer DNA-1000 Lab Chip (Agilent Technologies, 5067–1504, Santa Clara, CA, USA), and its concentration was measured using the Qubit dsDNA HS assay (Thermo Fisher Scientific, Q32854, Waltham, MA, USA). Sequencing was performed on a MiSeq next-generation sequencing (NGS) system (Illumina, San Diego, CA, USA) with 500 cycle v2-chemistry, generating 2 × 250 bp paired-end reads.

The sequencing library construction and Illumina MiSeq sequencing were completed by GENEWIZ Company (Suzhou, China). A Qubit 2.0 fluorometer (Invitrogen, Carlsbad, CA, USA) was used to detect the concentration of the DNA samples. The sequencing libraries were constructed using a MetaVx^TM^ Library Construction kit (GENEWIZ, Inc., South Plainfield, NJ, USA). 16S rDNA (30–50 ng) was amplified using an upstream primer (CCTACGGRRBGCASCAGKVRVGAAT) and a downstream primer (GGACTACNVGGGTWTCTAATCC) targeting the V3–V4 variable region. Indexes were added to the ends of the amplified products of 16S rDNA by PCR for NGS. The integrity of the library was evaluated on a Bioanalyzer Agilent 2100 (Agilent Technologies, 5067–1504, Palo Alto, CA, USA). The library concentration was measured using a Qubit 2.0 fluorometer (Invitrogen, Carlsbad, CA, USA). After mixing the DNA libraries, PE250 double-ended sequencing was performed according to the Illumina MiSeq (Illumina, San Diego, CA, USA) instructions. The sequence information was read by MiSeq Control Software (MCS), which was preinstalled on the MiSeq system. The sequencing run performed well, with 81.51% of all bases having a Q-score > 30. Paired-end reads were merged and quality trimmed, and primer sequences were removed. Merged sequences containing Ns were filtered, and sequences with a length greater than 200 bp were retained. After quality filtering, chimaeric sequences were removed, and the final sequence was used for operational taxonomic unit (OTU) analysis. VSEARCH (1.9.6) was used for sequence clustering (sequence similarity was set to 97%). The 16S rRNA reference database used for alignment was Silva 132. The RDP classifier (Ribosomal Database Program) Bayesian algorithm was used to analyse the representative sequences of OTUs, and the community composition of each sample was counted at different taxonomic levels. Based on the results of the OTU analysis, the abundance coverage estimator (ACE), Chao1, Shannon and Simpson alpha diversity indices were calculated by random sampling, and rarefaction curves were generated. Weighted UniFrac analysis was used to compare significant differences in microbial communities among samples. Based on the Bray-Curtis sample spacing matrix, a visual map of the principal coordinate analysis (PCoA) and nonmetric multidimensional scaling (NMDS) were used to show beta diversity. Redundancy analysis (RDA) was conducted based on the Hellinger method. Linear discriminant analysis (LDA) effect size (LEfSe) analysis was conducted by the lefse module on the online galaxy website.

### 2.4. Quantitative Reverse Transcription-PCR (qRT-PCR) to Assess Drosophila Gene Expression

Flies fed an inulin-supplemented or standard diet for twenty days were selected. Whole bodies (15–20 flies per sample) were homogenized in Buffer TLB (BioBase, N1002-01) using a tissue grinder (Tiangen, OSE-Y20) with a pestle (Tiangen, OSE-Y001). RNA concentrations were measured using the Nanodrop 2000 spectrophotometer (NanoDrop2000c; Thermo Scientific, Waltham, MA, USA), and 1 μg of total RNA per sample was reverse-transcribed using the FastKing First-Strand Synthesis system (Tiangen, KR116-02). qRT-PCR was performed using a Rotor-Gene 3000 real-time PCR cycler (Corbett/Qiagen, Hilden, Germany) with QuantiNova SYBR Green I PCR Master Mix (Qiagen, 208052, Dusseldorf, Germany). The final mRNA expression fold change relative to the control was normalized to rp49. Primer sequences for qRT-PCR are shown in Supplementary Table S1.

### 2.5. Statistics

Survival curvs and Mantel-Cox tests were analysed by GraphPad Prism 7 software (GraphPad Software, La Jolla, CA, USA). For other comparisons between two samples, two-tailed Student’s *t*-tests were used. For multiple comparisons, one-way analysis of variance with Tukey’s test was used. All graphs show the mean with error bars of 1 SD. Statistical significance is indicated by asterisks, where * *p* < 0.05, ** *p* < 0.01.

## 3. Results

### 3.1. Inulin Extends the InR^p5545^/TM3 D. melanogaster Lifespan in Males but Not in Females

**Figure 1:**
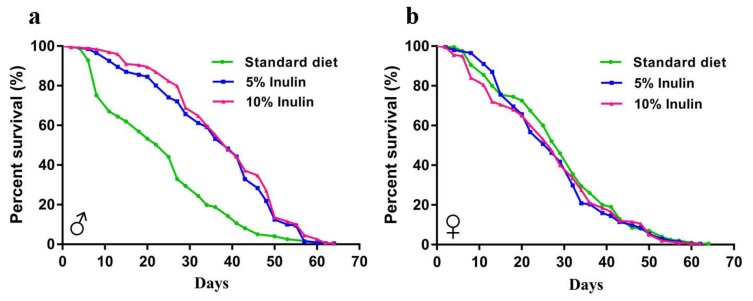
Lifespan analysis of *InR*^p5545^/TM3 *D. melanogaster* fed an inulin-supplemented diet or a standard diet for male (**a**) and female (**b**) flies.

First, we tested whether the lifespan of *InR*^p5545^/TM3 *D. melanogaster* was affected by inulin. The *InR*^p5545^/TM3 *D. melanogaster* lifespan was dramatically extended in the male group (Figure 1a); however, inulin did not increase the lifespan in the female group (Figure 1b). This finding was supported by the log-rank (Mantel-Cox) tests for survival curves (Table 1), which demonstrated a promotion of longevity by both 5% inulin (*p* < 0.0001) and 10% inulin (*p* < 0.0001) in male flies. However, neither 5% inulin (*p* = 0.3001) nor 10% inulin (*p* = 0.2570) affected the lifespan of flies in the female group. The mean lifespans of male flies were enhanced by 53.83% and 61.62% in the 5% and 10% inulin treatment groups, respectively, compared to that in the standard diet group.

### 3.2. Inulin Modulates InR^p5545^/TM3 D. melanogaster Metabolism

Considering the significant difference in lifespan extension in the male and female groups, we next tested whether the metabolic characteristics differed between the different genders under inulin treatment.

**Figure 2:**
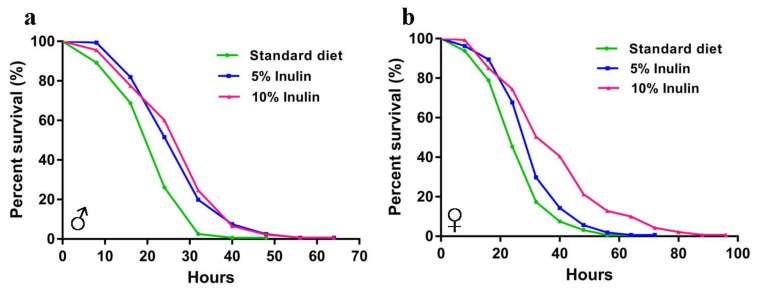
Starvation survival curves of *InR*^p5545^/TM3 *D. melanogaster* fed an inulin-supplemented diet or a standard diet for male (**a**) and female (**b**) flies.

**Figure 3:**
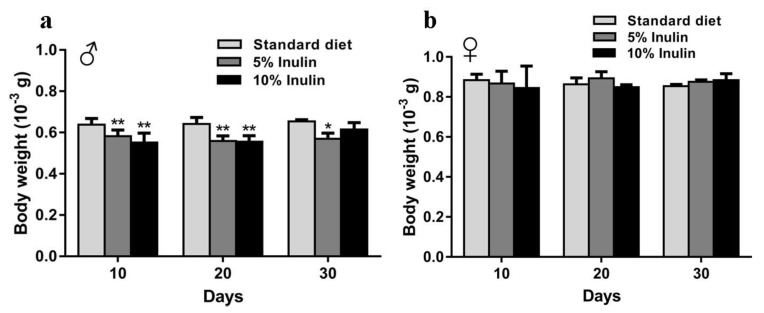
Body weight of *InR*^p5545^/TM3 *D. melanogaster* fed an inulin-supplemented diet or a standard diet in male (**a**) and female (**b**) flies. Error bars indicate standard errors. *p* values were calculated by comparing the inulin-fed flies to flies fed a nonsupplemented standard diet. * *p* ≤ 0.05; ** *p* < 0.01.

The results of starvation tests demonstrated that starvation resistance in both male and female *InR*^p5545^/TM3 *D. melanogaster* was improved by inulin (Figure 2). The log-rank (Mantel-Cox) tests showed that inulin significantly extended the mean starvation lifespan in both the male and female groups (Table 2, *p* < 0.0001, *p* = 0.0003). Changes in body weight were significantly decreased in male *InR*^p5545^/TM3 *D. melanogaster* supplemented with inulin on day 10, day 20, and day 30 (Figure 3a); however, the body weights of female flies were not significantly affected by inulin (Figure 3b).

### 3.3. Different Gene Expression Patterns in InR^p5545^/TM3 Males and Females Fed with Inulin

The different lifespan and body weight patterns in the male and female groups indicate that different underlying gene expression patterns may exist between the male and female groups, although both groups were supplemented with the same inulin. Therefore, we examined the above hypothesis by detecting the expression of related metabolism-, stress-, immune-, and longevity-associated genes in the inulin-supplemented male and female groups.

**Figure 4:**
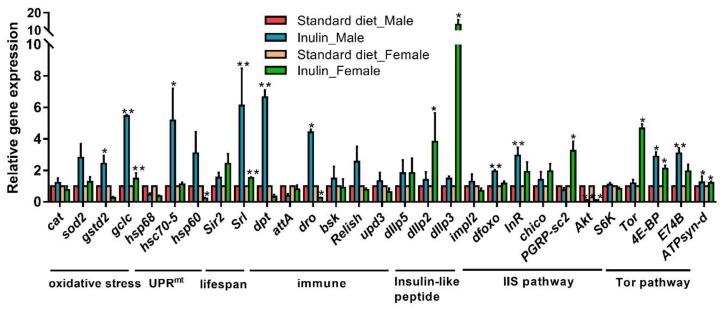
Effect of inulin on the relative transcript levels of metabolism-, stress-, immune-, and longevity-associated genes. * *p* ≤ 0.05; ** *p* < 0.01.

Intriguingly, *InR* was significantly upregulated in the inulin-supplemented male group; however, it was not affected by inulin in the female group (Figure 4). PGRP-sc2, another FOXO target gene, was significantly upregulated in female flies (Figure 4). However, the insulin receptor substrate Chico was not affected by inulin in either the male or female groups (Figure 4). The IIS pathway transcription factor Foxo was upregulated in the male group (Figure 4), and Akt was downregulated by inulin in both the male and female groups (Figure 4). Interestingly, dIlp2, dIlp3, and dIlp5, three insulin-like peptides, were unaffected by inulin in male flies; however, dIlp3 and dIlp5 in females were significantly upregulated by inulin (Figure 4). For the TOR pathway, 4E-BP was significantly affected by inulin in both the male and female groups, whereas TOR was significantly upregulated by inulin in the female group (Figure 4).

The mitochondrial unfolded protein response (UPR^mt^) gene hsc70-5 was upregulated in the male group; however, hsp60 was significantly downregulated in female flies (Figure 4). Srl, a lifespan-related gene, was significantly enhanced in both the male and female groups (Figure 4). For the seven tested immune-related genes in this study, only dpt and dro were upregulated in the male group, whereas dro was significantly downregulated in females (Figure 4). Among oxidative stress-related genes, gstd2 and gclc were significantly improved in the male group, and only gclc was upregulated in the female group (Figure 4). ATPsyn-d was significantly upregulated in both males and females, which may indicate that energy metabolism was improved by inulin.

### 3.4. Gut Microbiota

A total of 806,474 high-quality sequences were generated via sequencing, and a 97% identity cut-off was used to define each OTU by clustering to the reference sequences. The OTU numbers ranged from 20 to 27 for each sample. The 16S sequence data generated in this study were submitted to the NCBI SRA database (accession number PRJNA516993).

**Figure 5:**
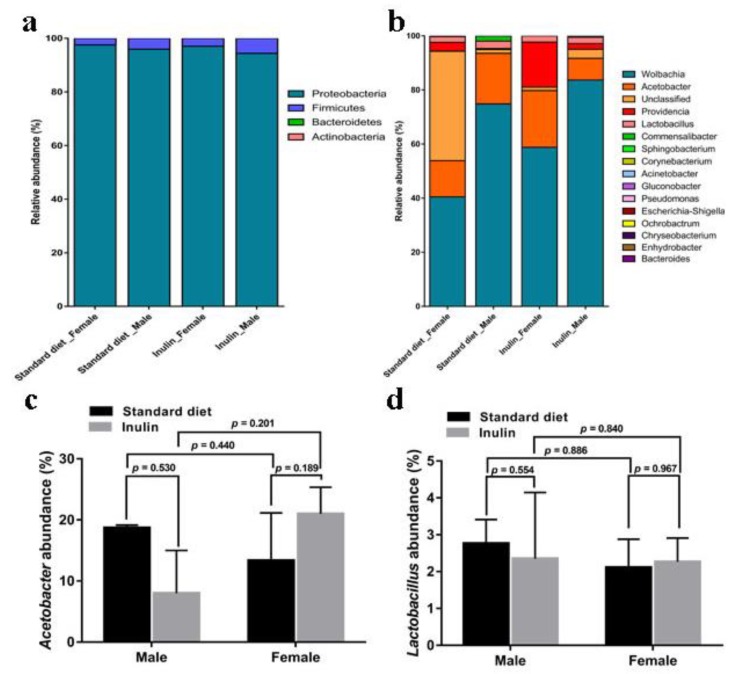
Compositions of gut microbiota for *InR*^p5545^/TM3 *D. melanogaster* fed an inulin-supplemented diet or a standard diet. Bacterial diversity at the phylum level (**a**) and genus level (**b**). Bacterial abundance of *Acetobacter* (**c**) and *Lactobacillus* (**d**).

As shown in Figure 5a, the bacteria in the *InR*^p5545^/TM3 *D. melanogaster* gut were mainly composed of the phyla Proteobacteria and Firmicutes, among which Proteobacteria was the most abundant phylum, accounting for over 90%. At the genus level, the largest component was Wolbachia, accounting for 40–80%, followed by Acetobacter, Providencia and Lactobacillus (Figure 5b). Acetobacter and Lactobacillus were considered the two major genera in Drosophila, and longevity was related to changes in the abundance of the two genera [19]. In our study, no change in the abundance of Acetobacter by inulin was observed in either male (*p* = 0.530) or female (*p* = 0.189) flies (Figure 5c), and similar results were found for Lactobacillus (Figure 5d). The abundances of Acetobacter (Figure 5c) and Lactobacillus (Figure 5d) were not significantly different between the male and female groups fed a standard diet. After inulin intervention, the Acetobacter and Lactobacillus abundance did not differ between male and female flies being fed the inulin-supplemented diet (Figure 5).

Species richness was measured by both the Chao1 estimator and ACE, and species evenness was expressed by the Shannon and Simpson estimators in our study. Richness was not significantly different between the male and female flies on a standard diet (p_Ace_ = 0.966, p_Chao1_ = 0.949), while the evenness was significantly different between the male and female flies on a standard diet (p_Simpson_ = 0.013). Inulin supplementation did not change the gut bacteria richness in either the male or female groups (Table 3), while the evenness between the male and female groups did not change from divergence to convergence after the inulin intervention.

**Figure 6:**
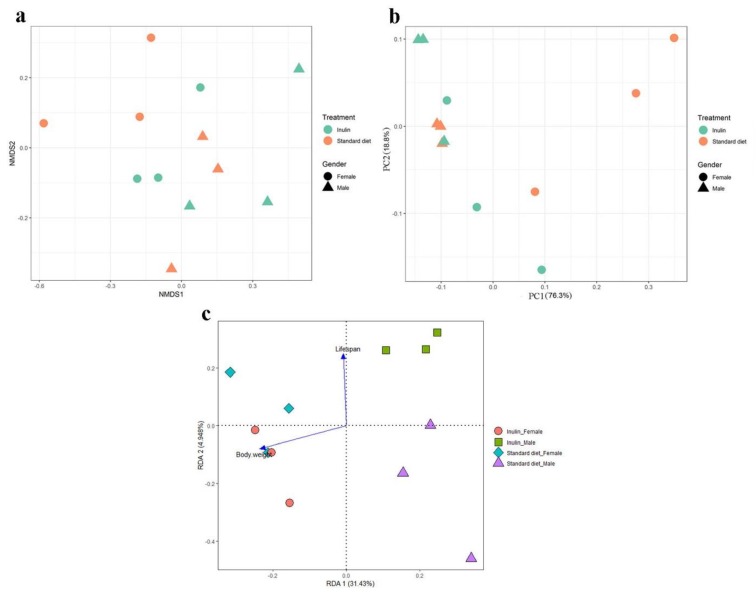
Nonmetric multidimensional scaling (NMDS) (**a**) principal coordinate analysis (PCoA) score plots (**b**) based on weighted UniFrac metrics and redundancy analysis (RDA) (**c**).

The NMDS results showed distinct patterns between males and females (Figure 6a), i.e., a relatively different gut microbiota composition between flies on the standard diet and those receiving the inulin intervention. An analysis of weighted UniFrac metrics (PCoA) showed that in the gut microbiota, the explained variance in PC1 was 76.3%, and the explained variance in PC2 was 18.8%, with no significant distinct separation between the inulin-supplemented group and the standard diet group, while a relatively distinct cluster distinguished the male and female groups (Figure 6b). In addition, the results of RDA showed that male flies on an inulin-supplemented diet showed a stronger relationship between lifespan and the gut microbiota than females (Figure 6c). Body weight showed a similar relationship with the gut microbiota between female flies on the standard diet and those on the inulin-supplemented diet and had a larger effect on the gut microbiota of male flies on the inulin-supplemented diet than on that of male flies on the standard diet (Figure 6c).

**Figure 7:**
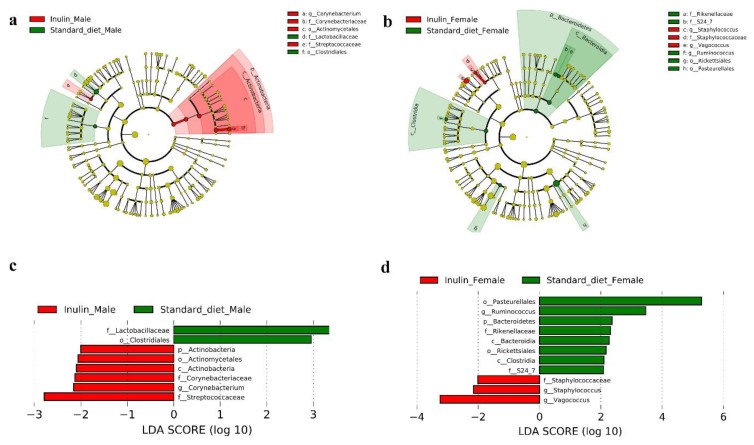
Cladogram and linear discriminant analysis (LDA) scores indicating significant differences in gut microbiota in *InR*^p5545^/TM3 *D. melanogaster* fed an inulin-supplemented diet or a standard diet. (**a**) Cladogram of male flies on the inulin and standard diet; (**b**) Cladogram of male and female flies on the inulin-supplemented diet; (**c**) LDA scores of male flies on the inulin and standard diet; (**d**) LDA scores of male and female flies on the inulin-supplemented diet.

LEfSe analysis was used to identify overrepresented OTUs and to compare their relative abundances between male flies on the standard and inulin-supplemented diets and between males and females on the inulin-supplemented diet. As shown in Figure 7a,b, clearly different evolutionary clusters were found between the two sex groups on the standard and inulin-supplemented diets. Among the significant beneficial microbiota, Lactobacillaceae was found to play a crucial role in males on the standard diet, while Streptococcaceae was enriched in males in the inulin-supplemented diet group (Figure 7c). However, among the beneficial microbiota in females, only Bacteroidetes was significantly enriched in flies on the standard diet, and none were enriched in flies in the inulin-supplemented diet group (Figure 7d).

## 4. Discussion

In this study, we attempted to assess the effects of inulin on the lifespan, related gene expression and gut community of *InR^p5545^*/TM3 *D. melanogaster*. The results showed that the lifespan was extended by inulin in only *InR^p5545^*/TM3 males and not in females. The gender-dependent lifespan promotion by dietary supplementation was also reported in previous studies. Both Wang et al. [20] and Zou et al. [21] reported that resveratrol extended the lifespan of female flies, although it did not affect male flies, and the authors suggested that female flies may have a strengthened response to resveratrol compared with that of males. Olga Boyd et al. [22] showed that the mean lifespan of female flies was significantly increased by 4% nectarine, while no significant change was observed in males. Schriner et al. [23] proved that cinnamon extended the lifespan of male *Drosophila* but not females. The above evidence indicates that the occurrence of gender-dependent responses is complicated and may be caused by many factors, e.g., different genetic background stocks, dietary supplementations, and dosages. In addition, the lack of a wild-type control in this study makes it difficult to determine whether the gender-dependent longevity by inulin in males was due to a specific effect of an *InR* mutation or a general effect on *Drosophila*.

To explore the potential molecular mechanisms underlying this gender-dependent longevity further, we next tested whether different patterns of expression of the related genes exist in male and female flies. Ageing is a complicated progress and is affected by many factors [24]. The IIS pathway is reported to be a central regulator of longevity [25,26], and IIS pathway single-gene loss-of-function mutations were found to promote a long lifespan in *Drosophila* [17,27,28]. Previous studies demonstrated mechanisms underlying regulation of the adult *Drosophila* lifespan via IIS and interacting pathways typically via the following four mechanisms: (1) activation of dFOXO [29]; (2) the TOR pathway [30]; (3) DILPs [28]; and (4) the JNK pathway [31]. Regarding the first mechanism, in our study, upregulated expression of *foxo* in male flies and unchanged expression in female flies suggested that the activation of *foxo* may be a promotor of lifespan extension. This difference was also supported by a study stating that reduced insulin signalling induced longevity effects resulting from activation of the transcription factor Foxo [32]. With regard to the second mechanism, TOR signalling is considered an important mediator of ageing in *Drosophila* [30,33,34]. The TOR complex 1 effector S6K1 has been shown to give rise to negative feedback on IIS [35], and the reduced activity of S6K1 was found to extend the lifespan [30]. The unchanged *S6K* in our study indicated that S6K does little to promote longevity in male flies. Studies have also shown that 4E-BP, another TOR effector, extended the lifespan by enhancing mitochondrial activity in *Drosophila* [36,37], which was consistent with the increased levels of *4E-BP* and *hsc70-5* in our study. This result indicated that a stronger mitochondrial unfolded protein response resulting from 4E-BP activation may be another contributor to lifespan enhancement in male flies. Considering the third mechanism, Broughton et al. [28] argued that a reduction in the expression of *dilps* led to decreased insulin signalling, which resulted in an increased median lifespan and starvation resistance. Our study showed no expression changes in *dilp2*, *dilp3*, or *dilp5*, suggesting that these genes had little effect on the lifespan extension in males in our study. Regarding the fourth mechanism, in our study, *bsk* remained unchanged after inulin treatment, indicating that the effect of inulin on lifespan extension may not occur via the JNK pathway.

In addition to the four above mentioned mechanisms, the increase in *InR* mRNA levels may have improved insulin signalling deficiency to some extent in male flies supplemented with inulin, which also partially contributed to the longevity observed in the male group. In addition, a previous study by Schriner et al. [23] proved that cinnamon extended the *Drosophila* lifespan via a sex-specific dependence on *chico*. However, the mRNA level of *chico* remained unchanged in both male and female flies after inulin treatment in our study. From this result, we inferred that the gender-specific responses in our study were reliant on other mechanisms.

Free radical oxidative damage is described as a health risk, and cumulative free radical oxidative damage to macromolecules has been reported to result in ageing [38,39]. In our study, *gstd2* and *gclc* were upregulated in male flies, and only *gclc* was increased in females, indicating that inulin improved oxidative resistance in both genders but to different extents. This finding was consistent with a previous publication showing increased oxidative resistance in flies by supplementation with resveratrol [20]. However, another study reported that antioxidant enzymes would allow the organism to cope better under stressful conditions, whereas these enzymes are probably weakly related to the normal ageing process [39].

Inulin was also suggested to be beneficial to the gut-associated lymphoid tissue [40,41] and macrophage-dependent immune responses [42]. In our study, the immune system was found to be modified by inulin in the male groups because the mRNA levels of *dpt* and *dro* were upregulated in male flies, whereas they remained unchanged in the female group. The enhanced immune responses were consistent with those reported in previous studies in which immune parameters, including IL-10 production, natural killer cell cytotoxicity, and secretory IgA, were modulated by inulin [43,44]. Enhanced immune responses to nectarine [22] and resveratrol [45] in *Drosophila* were also found.

Regarding the above mentioned gene expression data, metabolism-, stress-, immune-, and longevity-associated genes showed larger expression changes in male flies than in female flies, especially those of the IIS and TOR pathways. However, whether the changes in these gene pathways were caused by only *InR* mutation should be further investigated. TM3, a balancer chromosome with inversion of *In(3LR)*, may result in several mutations due to the multiple chromosomal rearrangements, and it disrupts protein-coding genes in *Drosophila*, including *Glut4EF*, *FucTA*, *CG32206*, *Lrrk*, *CG14459* and *kek6* [46]. Therefore, further studies should include controls for mutations linked to the *InR* mutation in the strain used here.

As a fermentable fibre, inulin plays roles via the gut microbiota, and previous research has confirmed that gut microbiota can modulate the expression of host genes, including those involved in immune responses, tissue homeostasis, gut physiology and metabolism, in *Drosophila melanogaster* [47]. Therefore, we inferred that the abovementioned related genes that were observed to be up- or downregulated in our study may be derived from the gut microbiota community shift. Our NMDS and RDA results showed that the gut microbiota results were distinctly separated by gender in both the standard diet group and the inulin treatment group (Figure 6a) and that lifespan had a stronger relationship with the gut microbiota community in male flies than in females (Figure 6c). Wong et al. [48] identified a relationship between the microbiota and protein nutrition (particularly in females), lipid/carbohydrate storage, and energy storage. Specifically, the significance of *Drosophila*–microbial interaction differences between males and females has been emphasized, i.e., a sex-specific interaction existed between the microbiota and the host metabolism [48]. The gut microbiota data in our study provide evidence that the gut microbiota plays an important role in gender-dependent responses between inulin and lifespan.

*Acetobacter* and *Lactobacillus* were related to metabolism, immune response, and ageing in *Drosophila*. Germ-free *Drosophila* inoculated with *Acetobacter* species have been reported to have a lower adult triglyceride (TG) storage level [49,50], and *Acetobacter pomorum* has the largest effect on host responses, including growth, development, body size, and metabolism, by affecting the insulin signalling pathway [51]. Additionally, *Lactobacillus plantarum* was reported to increase the larval growth rate as a result of the increased release of insulin-like peptides by modulation of the TOR pathway [52]. In addition, the cell walls of *Acetobacter* and *Lactobacillus spp.* have been reported to contain DAP-type peptidoglycan, suggesting that these species can be recognized by the gut immune response [53]; they were also reported to have an effect on epithelium physiology [54]. Fumiaki et al. reported that the lifespan extension in *Drosophila* occurred via gut microbiome remodelling, in which *Acetobacter* was selectively depleted, while *Lactobacilli* was not [19]. However, in our study, inulin did not affect the *Acetobacter* abundance in comparison to that in the standard diet group nor did it influence the *Acetobacter* abundance between the male and female groups. Among the gut probiotics, Lactobacillaceae was found to play a crucial role in males on a standard diet, while Streptococcaceae was enriched in males on the inulin-supplemented diet group in our study, indicating that the gut probiotics are changed by inulin. Whether Streptococcaceae contributes to longevity in male flies requires further investigation. In brief, although the distinctly separated patterns of the gut microbiota community of the two genders in our study provided evidence for gender-dependent longevity, more direct evidence linking the gut microbiota to lifespan is needed.

We are obliged to address several limitations in the experimental design of this study. First, the balancer chromosome of TM3 contains multiple additional mutations to *InR*, so we cannot generalize our results beyond the strain used here without further studies controlling for genetic background. Second, it is difficult to determine whether gender-dependent longevity was a general effect or a specific effect of inulin because of the lack of a wild-type *Drosophila melanogaster*.

In summary, in this study, we observed a gender-dependent lifespan extension by inulin in *InR*^p5545^/TM3 *D. melanogaster* and distinctly different patterns in the related gene expression levels. Given the data on gene expression, we further speculate three possible mechanisms of longevity in *InR*^p5545^/TM3 *D. melanogaster* male flies: first, the activation of *foxo*; second, the regulation of lifespan by *4E-BP* by mediating the UPR^mt^ *hsc70-5* via the TOR pathway; third, the improvement of *InR* may regulate lifespan. However, whether these gene pathway changes resulted from the *InR* mutation or other mutations in the *InR*^p5545^/TM3 strain needs to be further verified. The gut microbiota community was distinctly separated by gender in both the standard diet group and the inulin treatment group, and the relationship between lifespan and the gut microbiota community was stronger in male flies than in females. Identifying genetic mechanisms mediating interactions between the gut microbiota and the inulin-dependent lifespan response would contribute to a deeper understanding of gut microbiota function and the role of gut-brain communication.

## Figures and Tables

**Table 1:** Statistics for survival curves. Cohort sizes, mean and median lifespans, percentage changes, and log-rank (Mantel-Cox) tests for survival curves in this study.

	Total n. of Flies	Mean (% Change)	Median (% Change)	Log-Rank (vs. Standard Diet)
**Male**				
Standard diet	196	34.91	22.08	-
5% Inulin	200	53.70 (+53.83%)	60.20 (+172.63%)	*p* < 0.0001
10% Inulin	198	56.42 (+61.62%)	62.31 (+182.19%)	*p* < 0.0001
**Female**				
Standard diet	199	42.60	32.50	-
5% Inulin	200	40.73 (−4.39%)	29.85 (−8.15%)	*p* = 0.3001
10% Inulin	199	39.75 (−6.69%)	33.50 (+3.08%)	*p* = 0.2570

**Table 2:** Statistics for starvation–survival curves. Cohort sizes, mean and median lifespans, percentage changes, and log-rank (Mantel-Cox) tests for starvation–survival curves in this study.

	Total n. of Flies	Mean (% Change)	Median (% Change)	Log-Rank (vs. Standard Diet)
**Male**				
Standard diet	156	28.79	14.33	-
5% Inulin	160	36.40 (+26.43%)	13.66 (−4.65%)	*p* < 0.0001
10% Inulin	137	36.81 (+27.87%)	15.58 (+8.71%)	*p* < 0.0001
**Female**				
Standard diet	160	24.80	12.42	-
5% Inulin	160	29.02 (+16.99%)	14.28 (+15.00%)	*p* = 0.0003
10% Inulin	140	35.82 (+44.41%)	17.02 (+37.02%)	*p* < 0.0001

**Table 3:** Alpha index analysis of *InR*^p5545^/TM3 *D. melanogaster* on an inulin-supplemented diet or a standard diet.

	Standard Diet Male	Standard Diet Female	Inulin Male	Inulin Female
ACE	25.74 ± 0.13	25.59 ± 4.68 (*p* = 0.966)	29.13 ± 2.74 (*p* = 0.155)	31.43 ± 9.24 (*p* = 0.753, 0.470)
Chao1	24.36 ± 0.69	24.17 ± 3.97 (*p* = 0.949)	33.67 ± 8.18 (*p* = 0.184)	25.72 ± 2.44 (*p* = 0.258, 0.661)
Shannon	1.40 ± 0.06	1.86 ± 0.29 (*p* = 0.094)	0.92 ± 0.36 (*p* = 0.135)	1.78 ± 0.40 (*p* = 0.087, 0.819)
Simpson	0.43 ± 0.02	0.64 ± 0.07 (*p* = 0.013)	0.28 ± 0.13 (*p* = 0.198)	0.57 ± 0.15 (*p* = 0.112, 0.601)

Note: Values are the mean ± SD (*n* = 3); the *p* value for the Standard diet Female was the Standard diet Female vs. Standard diet Male; the *p* value for Inulin Male was the Inulin Male vs. Standard diet Male; the first *p* value for Inulin Female was the Inulin Female vs. Inulin Male; and the second *p* value for Inulin Female was the Inulin Female vs. Standard diet Female.

## Supplementary Materials

The following are available online at https://www.mdpi.com/2072-6643/11/3/636/s1, Table S1: Primer sequences used for qRT-PCR in this study.

## References

[1] Lillycrop K.A. (2011). Effect of maternal diet on the epigenome: Implications for human metabolic disease. Proc. Nutr. Soc..

[2] Lee H.-S. (2015). Impact of maternal diet on the epigenome during in utero life and the developmental programming of diseases in childhood and adulthood. Nutrients.

[3] Zheng H., Zhang C., Yang W., Wang Y., Lin Y., Yang P., Yu Q., Fan J., Liu E. (2009). Fat and cholesterol diet induced lipid metabolic disorders and insulin resistance in rabbit. Exp. Clin. Endocrinol. Diabetes.

[4] Feige J.N., Lagouge M., Canto C., Strehle A., Houten S.M., Milne J.C., Lambert P.D., Mataki C., Elliott P.J., Auwerx J. (2008). Specific SIRT1 activation mimics low energy levels and protects against diet-induced metabolic disorders by enhancing fat oxidation. Cell Metab..

[5] Cai D., Zhao S., Li D., Chang F., Tian X., Huang G., Zhu Z., Liu D., Dou X., Li S. (2016). Nutrient Intake Is associated with longevity characterization by metabolites and element profiles of healthy centenarians. Nutrients.

[6] Hur K.Y., Lee M.-S. (2015). Gut microbiota and metabolic disorders. Diabetes Metab. J..

[7] Delzenne N.M., Neyrinck A.M., Bäckhed F., Cani P.D. (2011). Targeting gut microbiota in obesity: Effects of prebiotics and probiotics. Nat. Rev. Endocrinol..

[8] Round J.L., Mazmanian S.K. (2009). The gut microbiota shapes intestinal immune responses during health and disease. Nat. Rev. Immunol..

[9] Gibson G.R., Beatty E.R., Wang X., Cummings J.H. (1995). Selective stimulation of bifidobacteria in the human colon by oligofructose and inulin. Gastroenterology.

[10] Niness K.R. (1999). Inulin and Oligofructose: What Are They?. J. Nutr..

[11] Tarini J., Wolever T.M.S. (2010). The fermentable fibre inulin increases postprandial serum short-chain fatty acids and reduces free-fatty acids and ghrelin in healthy subjects. Appl. Physiol. Nutr. Metab..

[12] Kolida S., Tuohy K., Gibson G.R. (2007). Prebiotic effects of inulin and oligofructose. Br. J. Nutr..

[13] Zhao L., Zhang F., Ding X., Wu G., Lam Y.Y., Wang X., Fu H., Xue X., Lu C., Ma J. (2018). Gut bacteria selectively promoted by dietary fibers alleviate type 2 diabetes. Science.

[14] Koh A., De Vadder F., Kovatcheva-Datchary P., Backhed F. (2016). From dietary fiber to host physiology: Short-chain fatty acids as key bacterial metabolites. Cell.

[15] Delzenne N.M., Cani P.D., Daubioul C., Neyrinck A.M. (2007). Impact of inulin and oligofructose on gastrointestinal peptides. Br. J. Nutr..

[16] Baker K.D., Thummel C.S. (2007). Diabetic larvae and obese flies—Emerging studies of metabolism in *Drosophila*. Cell Metab..

[17] Tatar M., Kopelman A., Epstein D., Tu M.-P., Yin C.-M., Garofalo R.S. (2001). A mutant *Drosophila* insulin receptor homolog that extends life-span and impairs neuroendocrine function. Science.

[18] Patterson J.K., Yasuda K., Welch R.M., Miller D.D., Lei X.G. (2010). Supplemental dietary inulin of variable chain lengths alters intestinal bacterial populations in young pigs. J. Nutr..

[19] Obata F., Fons C.O., Gould A.P. (2018). Early-life exposure to low-dose oxidants can increase longevity via microbiome remodelling in *Drosophila*. Nat. Commun..

[20] Wang C., Wheeler C.T., Alberico T., Sun X., Seeberger J., Laslo M., Spangler E., Kern B., de Cabo R., Zou S. (2013). The effect of resveratrol on lifespan depends on both gender and dietary nutrient composition in Drosophila melanogaster. AGE.

[21] Zou S., Carey J.R., Liedo P., Ingram D.K., Müller H.-G., Wang J.-L., Yao F., Yu B., Zhou A. (2009). The prolongevity effect of resveratrol depends on dietary composition and calorie intake in a tephritid fruit fly. Exp. Gerontol..

[22] Boyd O., Weng P., Sun X., Alberico T., Laslo M., Obenland D.M., Kern B., Zou S. (2011). Nectarine promotes longevity in *Drosophila melanogaster*. Free Radic. Biol. Med..

[23] Schriner S.E., Kuramada S., Lopez T.E., Truong S., Pham A., Jafari M. (2014). Extension of *Drosophila* lifespan by cinnamon through a sex-specific dependence on the insulin receptor substrate *chico*. Exp. Gerontol..

[24] Kenyon C.J. (2010). The genetics of ageing. Nature.

[25] Barzilai N., Huffman D.M., Muzumdar R.H., Bartke A. (2012). The critical role of metabolic pathways in aging. Diabetes.

[26] Broughton S., Partridge L. (2009). Insulin/IGF-like signalling, the central nervous system and aging. Biochem. J..

[27] Clancy D.J., Gems D., Harshman L.G., Oldham S., Stocker H., Hafen E., Leevers S.J., Partridge L. (2001). Extension of life-span by loss of CHICO, a Drosophila insulin receptor substrate protein. Science.

[28] Broughton S.J., Piper M.D.W., Ikeya T., Bass T.M., Jacobson J., Driege Y., Martinez P., Hafen E., Withers D.J., Leevers S.J. (2005). Longer lifespan, altered metabolism, and stress resistance in *Drosophila* from ablation of cells making insulin-like ligands. Proc. Natl. Acad. Sci. USA.

[29] Giannakou M.E., Goss M., Junger M.A., Hafen E., Leevers S.J., Partridge L. (2004). Long-lived *Drosophila* with overexpressed dFOXO in adult fat body. Sci. Aging Knowl. Environ..

[30] Kapahi P., Zid B.M., Harper T., Koslover D., Sapin V., Benzer S. (2004). Regulation of lifespan in *Drosophila* by modulation of genes in the TOR signaling pathway. Curr. Biol..

[31] Wang M.C., Bohmann D., Jasper H. (2005). JNK extends life span and limits growth by antagonizing cellular and organism-wide responses to insulin signaling. Cell.

[32] Wang L., Karpac J., Jasper H. (2014). Promoting longevity by maintaining metabolic and proliferative homeostasis. J. Exp. Biol..

[33] Katewa S.D., Kapahi P. (2011). Role of TOR signaling in aging and related biological processes in *Drosophila melanogaster*. Exp. Gerontol..

[34] Partridge L., Alic N., Bjedov I., Piper M.D.W. (2011). Ageing in *Drosophila*: The role of the insulin/Igf and TOR signalling network. Exp. Gerontol..

[35] Kockel L., Kerr K.S., Melnick M., Brückner K., Hebrok M., Perrimon N. (2010). Dynamic switch of negative feedback regulation in *Drosophila* Akt–Tor signaling. PLOS Genet..

[36] Zid B.M., Rogers A.N., Katewa S.D., Vargas M.A., Kolipinski M.C., Lu T.A., Benzer S., Kapahi P. (2009). 4E-BP extends lifespan upon dietary restriction by enhancing mitochondrial activity in *Drosophila*. Cell.

[37] Morita M., Gravel S.-P., Chénard V., Sikström K., Zheng L., Alain T., Gandin V., Avizonis D., Arguello M., Zakaria C. (2013). mTORC1 controls mitochondrial activity and biogenesis through 4E-BP-dependent translational regulation. Cell Metab..

[38] Finkel T., Holbrook N.J. (2000). Oxidants, oxidative stress and the biology of ageing. Nature.

[39] Le Bourg É. (2001). Oxidative stress, aging and longevity in *Drosophila melanogaster*. FEBS Lett..

[40] Seifert S., Watzl B. (2007). Inulin and oligofructose: Review of experimental data on immune modulation. J. Nutr..

[41] Roller M., Rechkemmer G., Watzl B. (2004). Prebiotic inulin enriched with oligofructose in combination with the probiotics *Lactobacillus rhamnosus* and *Bifidobacterium lactis* modulates intestinal immune functions in rats. J. Nutr..

[42] Kelly-Quagliana K.A., Nelson P.D., Buddington R.K. (2003). Dietary oligofructose and inulin modulate immune functions in mice. Nutr. Res..

[43] Watzl B., Girrbach S., Roller M. (2007). Inulin, oligofructose and immunomodulation. Br. J. Nutr..

[44] Gourbeyre P., Desbuards N., Grémy G., Le Gall S., Champ M., Denery-Papini S., Bodinier M. (2012). Exposure to a galactooligosaccharides/inulin prebiotic mix at different developmental time points differentially modulates immune responses in mice. J. Agric. Food Chem..

[45] Baur J.A., Pearson K.J., Price N.L., Jamieson H.A., Lerin C., Kalra A., Prabhu V.V., Allard J.S., Lopez-Lluch G., Lewis K. (2006). Resveratrol improves health and survival of mice on a high-calorie diet. Nature.

[46] Miller D.E., Cook K.R., Arvanitakis A.V., Hawley R.S. (2016). Third chromosome balancer inversions disrupt protein-coding genes and influence distal recombination events in *Drosophila melanogaster*. G3.

[47] Broderick N.A., Buchon N., Lemaitre B. (2014). Microbiota-induced changes in *Drosophila melanogaster* host gene expression and gut morphology. mBio.

[48] Wong A.C.-N., Dobson A.J., Douglas A.E. (2014). Gut microbiota dictates the metabolic response of *Drosophila* to diet. J. Exp. Biol..

[49] Chaston J.M., Newell P.D., Douglas A.E. (2014). Metagenome-wide association of microbial determinants of host phenotype in *Drosophila melanogaster*. mBio.

[50] Huang J.-H., Douglas Angela E. (2015). Consumption of dietary sugar by gut bacteria determines *Drosophila* lipid content. Biol. Lett..

[51] Shin S.C., Kim S.-H., You H., Kim B., Kim A.C., Lee K.-A., Yoon J.-H., Ryu J.-H., Lee W.-J. (2011). *Drosophila* microbiome modulates host developmental and metabolic homeostasis via insulin signaling. Science.

[52] Storelli G., Defaye A., Erkosar B., Hols P., Royet J., Leulier F. (2011). *Lactobacillus plantarum* promotes *Drosophila* systemic growth by modulating hormonal signals through tor-dependent nutrient sensing. Cell Metab..

[53] Ryu J.-H., Kim S.-H., Lee H.-Y., Bai J.Y., Nam Y.-D., Bae J.-W., Lee D.G., Shin S.C., Ha E.-M., Lee W.-J. (2008). Innate immune homeostasis by the homeobox gene *Caudal* and commensal-gut mutualism in *Drosophila*. Science.

[54] Erkosar B., Leulier F. (2014). Transient adult microbiota, gut homeostasis and longevity: Novel insights from the *Drosophila* model. FEBS Lett..

