# Supplementary material for: The Effect of Inulin on Lifespan, Related Gene Expression and Gut Microbiota in InRp5545/TM3 Mutant Drosophila melanogaster: A Preliminary Study
Source: Nutrients. 2019 Mar 15;11(3):636. doi: 10.3390/nu11030636 (PMC6470987; doi:10.3390/nu11030636)
Supplement: Supplementary file 1 [file nutrients-11-00636-s001.pdf]

Table S1. Primer Sequences of qRT-PCR used in this study

Gene	Primer sequences (5'→3')	Reference
cat	CCTCTGATTCCTGTGGGCAA; GACGACCATGCAGCATCTTG	[1]
sod2	AATTTCGCAAACTGCAAGC; TGATGCAGCTCCATGATCTC	[2]
gstd2	CTCCAATGTCTCCAGGTGGT; CCCAGTTCTCATCCCATCC	[2]
gclc	CGAGGAGAATGAGCTGTTCC; ACCAGACCCGGAAAAACG	[2]
hsp68	GGAGGCTCCACTCGTATTCC; TCTTTCCGCCGAAGAAGTT	[3]
hsc70-5	GGAATTGATATCCGCAAGGA; TCAGCTTCAGGTTCATGTGC	[2]
hsp60	TGATGCTGATCTCGTCAAGC; TACTCGGAGGTGGTGTCCTC	[2]
Sir2	CCGTTACTGAGGAGGAGCTG; GTAGATCGCACACGTCCTTG	[4]
Srl	CTCTTGGAGTCCGAGATCCGCAA; GGGACCGCGAGCTGATGGTT	[4]
dpt	GTTCACCATTGCCGTCGCCTTAC; CCCAAGTGCTGTCCATATCCTCC	[2]
attA	CACAATGTGGTGGGTCAGG; GGCACCATGACCAGCATT	[2]
dro	CCATCGAGGATCACCTGACT; CTTTAGGCGGGCAGAATG	[2]
bsk	CACTCAGCAGGAATTATTCACAGA; TTAGAGTGCAGTCGGCCTTT	[3]
Relish	GACCCGAAAGCTCGGCGCAAA; TCGCTCACGAGTTGCGAGCAA	[5]
Spn43Aa	TGATCATCTCGCCGGTTTC; CAGACCATCTTTGCTCTCCTT	[6]
upd3	ACTGGGAGAACACCTGCAAT; GCCCGTTTGGTTCTGTAGAT	[7]
dIlp5	CAAACGAGGCACCTTGGG; AGCTATCCAAATCCGCCA	[3]
dIlp2	ATCCCGTGATTCCACACAAG; GCGGTTCCGATATCGAGTTA	[3]
dIlp3	CCGAAACTCTCTCCAAGCTC; GCCATCGATCTGATTGAAGTT	[3]
E74B	GAATCCGTAGCCTCCGACTGT; AGGAGGGAGAGTGGTGGTGTT	[2]
impl2	GCCGATACCTTCGTGTATCC; TTTCCGTCGTCAATCCAATAG	[2]
dfoxo	TCGAGTGCAATGTCGAGGAG; AGCGGTATATTGATGTCCAGCAG	[3]
InR	AACAGTGGCGGATTCGGTT; TACTCGGAGCATTGGAGGCAT	[2]
PGRP-sc2	CCAAGTCTATCGGCATCTCC; GAGCAGAGGTGAGGGTGTTG	[2]
S6K	TGACCTAGAACCGGAATTGTG; TCCTCGCAGAGCTGTATGG	[3]
Tor	GCTCAGAGGCGAGAGACAAG; CCAGCTCACGGAGGATAAAG	[3]
4E-BP	CCAGATGCCCGAGGTGTA; AGCCCGCTCGTAGATAAGTTT	[3]
chico	GGCATACGGGCAGCTAGAC; TTCTTGAGGTAGCCACTCAGC	[3]
Akt	GAGTCGTGTGCTCAAGTCCA; TGCATCACAAAACACAGGCG	[8]
Rp49	GACGCTTCAAGGGACAGTATCTG; AAACGCGGTTCTGCATGA	[9]

References

1. Staats, S.; Wagner, A.; Kowalewski, B.; Rieck, F.; Soukup, S.; Kulling, S.; Rimbach, G., Dietary resveratrol does not affect life span, body composition, stress response, and longevity-related gene expression in *Drosophila melanogaster*. *International Journal of Molecular Sciences* **2018,** 19, (1), 223.
2. Obata, F.; Fons, C. O.; Gould, A. P., Early-life exposure to low-dose oxidants can increase longevity via microbiome remodelling in *Drosophila*. *Nature Communications* **2018,** 9, (1), 975.
3. Boyd, O.; Weng, P.; Sun, X.; Alberico, T.; Laslo, M.; Obenland, D. M.; Kern, B.; Zou, S.,

Nectarine promotes longevity in *Drosophila melanogaster*. *Free Radical Biology and Medicine* **2011,** 50, (11), 1669-1678.

4. Tinkerhess, M. J.; Healy, L.; Morgan, M.; Sujkowski, A.; Matthys, E.; Zheng, L.; Wessells, R. J., The *Drosophila* PGC-1α homolog spargel modulates the physiological effects of endurance exercise. *PLOS ONE* **2012,** 7, (2), e31633.
5. Bandarra, D.; Biddlestone, J.; Mudie, S.; Muller, H. A.; Rocha, S., Hypoxia activates IKK–NF-κB and the immune response in *Drosophila melanogaster*. *Bioscience Reports* **2014,** 34, (4).
6. Seong, K. M.; Coates, B. S.; Sun, W.; Clark, J. M.; Pittendrigh, B. R., Changes in neuronal signaling and cell stress response pathways are associated with a multigenic response of *Drosophila melanogaster* to ddt selection. *Genome Biology and Evolution* **2017,** 9, (12), 3356-3372.
7. Woodcock, Katie J.; Kierdorf, K.; Pouchelon, Clara A.; Vivancos, V.; Dionne, Marc S.; Geissmann, F., Macrophage-derived upd3 cytokine causes impaired glucose homeostasis and reduced lifespan in *Drosophila* fed a lipid-rich diet. *Immunity* **2015,** 42, (1), 133-144.
8. Westfall, S.; Lomis, N.; Prakash, S., Longevity extension in *Drosophila* through gut-brain communication. *Scientific Reports* **2018,** 8, (1), 8362.
9. Storelli, G.; Defaye, A.; Erkosar, B.; Hols, P.; Royet, J.; Leulier, F., *Lactobacillus plantarum* promotes *Drosophila* systemic growth by modulating hormonal signals through TOR-dependent nutrient sensing. *Cell Metabolism* **2011,** 14, (3), 403-414.
